# The progress in the field of clinical staging for mental disorders within the last decade: an updated systematic review

**DOI:** 10.3389/fpsyt.2024.1473051

**Published:** 2025-01-15

**Authors:** Sharon L. Clarke, Nicole Soons, Arjan C. Videler, Sebastiaan P. J. van Alphen, Henricus Van, Linda Dil, Laurens Pappijn, Sven Corbeij, Beau Broekhof, Andrew M. Chanen, Joost Hutsebaut

**Affiliations:** ^1^ Center of Research on Psychological Disorders and Somatic Diseases (CoRPS), Department of Medical and Clinical Psychology, Tilburg University, Tilburg, Netherlands; ^2^ Viersprong Institute for Studies on Personality Disorders, De Viersprong, Halsteren, Netherlands; ^3^ Clinical Centre of Excellence for Older Adults with Personality Disorders, Mondriaan Mental Health Center, Heerlen-Maastricht, Netherlands; ^4^ PersonaCura, Clinical Centre of Excellence for Personality and Developmental Disorders in Older Adults, GGZ Breburg, Tilburg, Netherlands; ^5^ Tranzo, Scientific Centre for Care and Wellbeing of the Tilburg School of Social and Behavioral Sciences of Tilburg University, Tilburg, Netherlands; ^6^ Personality and Psychopathology Research Group (PEPS), Department of Psychology, Vrije Universiteit Brussel (VUB), Brussels, Belgium; ^7^ NPI Centre for Personality Disorders, Arkin, Amsterdam, Netherlands; ^8^ Levvel, Amsterdam, Netherlands; ^9^ Orygen, Melbourne, VIC, Australia; ^10^ Centre for Youth Mental Health, The University of Melbourne, Melbourne, VIC, Australia

**Keywords:** staging, mental disorder, systematic review, clinimetrics, psychiatric disorder, psychiatry

## Abstract

**Introduction:**

Clinical staging aims to refine psychiatric diagnosis by describing mental disorders on a continuum of disorder progression, with the pragmatic goal of improved treatment planning and outcome prediction. The first systematic review on this topic, published a decade ago, included 78 papers, and identified separate staging models for schizophrenia, unipolar depression, bipolar disorder, panic disorder, substance use disorder, anorexia, and bulimia nervosa. The current review updates this review by including new proposals for staging models and by systematically reviewing research based upon full or partial staging models since 2012.

**Methods:**

PsycINFO, MEDLINE, EMBASE, and the Cochrane databases were systematically searched from 2012 to June 2023. The original review’s eligibility criteria were used and extended with newly introduced categories of DSM-5 mental disorders, along with mental disorders for which a progressive course might be expected. Included papers: a) contained a complete or partial staging model, or b) focused upon clinical features that might be associated with stages, or c) focused upon treatment research associated with specific stages.

**Results:**

Seventy-one publications met the inclusion criteria. They described staging models for schizophrenia and related psychoses (21 papers), bipolar (20), depressive (4), anxiety (2), obsessive-compulsive (3), trauma related (4), eating (3), personality disorders (2), and ‘transdiagnostic’ staging models (13).

**Discussion:**

There is a steady but slow increase in interest in clinical staging and evidence for the validity of staging remains scarce. Staging models might need to be better tailored to the complexities of mental disorders to improve their clinical utility.

**Systematic review registration:**

https://www.crd.york.ac.uk/prospero/, identifier CRD42021291703.

## 1 Introduction

Prevailing systems of psychiatric diagnosis have been widely criticized for not capturing the heterogeneity among individuals who meet the same diagnosis (1) and for lacking a perspective on the development and longitudinal course of a mental disorder, thereby failing to capture the dynamic nature of mental disorders (2). ‘Clinical staging’ aims to refine psychiatric diagnosis by describing mental disorders along an assumed continuum of disorder progression (3), across sequential stages describing progression of disorder processes. Originally developed in the field of oncology, staging models aim to enhance prognosis prediction and guide treatment decisions. The adoption of clinical staging in psychiatry was catalyzed by McGorry and colleagues’ model (4–6) and is intended to provide a more useful clinical framework for decisions about treatment assignment and the proportionality of such treatments to the presenting problems (3, 5). Initially proposed for psychotic disorders and later adapted for other diagnoses (e.g., bipolar, anxiety and depressive disorders), the model distinguishes five main stages (0-4), with stages 1 and 3 being further subdivided. Such a sequence of stages aligns with concepts of personalized or stratified care (7), enabling professionals to tailor treatment to the specific stage of a disease, called ‘staged care’ (8). Moreover, focusing clinical attention upon early stages of mental ill-health promotes a more hopeful, and potentially more effective, mental health care system, stressing the potential for prevention and early intervention for what might be seen to be, or experienced as, a progressive and/or severe mental illness (9). Staging models aim to optimize the timing of therapeutic interventions, embracing the assumption that the later stages of a disorder might be avoided or ameliorated by identifying and treating early precursors (6, 10).

One decade ago, Cosci and Fava (11) conducted the first systematic review of staging models in psychiatry. They identified 78 publications that met their inclusion criteria, including staging models for discrete disorders such as schizophrenia, unipolar depression, bipolar disorder, panic disorder, substance use disorder, anorexia nervosa, and bulimia nervosa. The current review aims to update this review. As the concept of staging was still relatively novel in 2012, it is relevant to review progress in the field and to what extent staging models have been empirically validated. Moreover, since the 2012 review, publication of the DSM-5 introduced some changes in psychiatric nosology. Finally, the original review did not contain all mental disorders for which a progressive course might be expected (e.g., personality disorders).

The first aim was to collect new proposals for full or partial staging models since 2012. Full staging models were defined as consisting of multiple stages, comprehensively describing the full course of a disorder, while a partial staging model describes only some stages. The second aim was to provide an overview of empirical papers substantiating the reliability, validity, and clinical utility of staging models.

## 2 Methods

The present systematic review followed the Preferred Reporting Items for Systematic Reviews and Meta-Analyses (PRISMA) Guidelines (12) and was preregistered at PROSPERO (ID: CRD42021291703).

### 2.1 Eligibility criteria

Papers eligible for screening were written in English, published in a peer-reviewed journal and reported data on humans with mental disorders according to the DSM-III (13), -IIIR (14), -IV (15), 1994), -IVTR (16), or -5 (17), the Research Diagnostic Criteria (18) or the International Classification of Diseases (19). We used the same criteria as Cosci and Fava (11), meaning that the following papers were eligible for screening: category a) papers wherein a (full or partial) staging model was proposed including a motivation of its existence; category b) papers studying clinical features related to a staging model; category c) papers studying treatment interventions related to a staging model. Additional inclusion criteria for category b) were: inclusion of at least 10 patients; papers with participants with co-occurring mental or organic disorders were also included. Additional inclusion criteria for category c) were: inclusion of at least 10 patients, inclusion of a comparison group or a crossover design, at least a double-blind design in the case of pharmacological treatments, at least a single-blind design in the case of nonpharmacological treatments. Papers with a primary focus on neuroanatomy or biological markers were excluded.

### 2.2 Information sources and searches

The search strategy of Cosci and Fava was replicated, including the databases used by Cosci and Fava, which were Medline, psychINFO, EMBASE and Cochrane. The databases were systematically searched from May 2012 to June 2023. Reference lists of relevant systematic reviews on clinical staging and of all included papers were checked for additional papers. Search terms were ‘stage OR stages OR staging’, combined using the Boolean ‘AND’ operator with different categories of mental disorders. Abstracts, titles, and keywords were searched. We combined with the following search terms: ‘mental disorder’, ‘psychiatric disorder’, ‘mood disorder’, ‘anxiety disorder’, ‘substance abuse disorder’, ‘schizophrenia’, ‘eating disorder’, ‘conduct disorder’, and ‘personality disorder’. These search terms deviated in two ways from Cosci and Fava’s (11) original search terms. First, we chose to include additional categories of mental disorders for which a potentially progressive course may be assumed, i.e., personality and conduct disorders. Second, we wanted to account for changes in the transition to DSM-5 and therefore performed an additional search including the search terms ‘obsessive compulsive disorders’ and ‘posttraumatic stress disorder’. In the **Supplementary Material** we reported the search strategy.

The searches in the databases were conducted by one reviewer (S.Cl.) and references were exported to Endnote (20). After removal of duplicates in Endnote (21), the remaining papers were exported to Rayyan (22). The last duplicates were removed by hand in Rayyan. Papers were screened by four independent reviewers (S.Cl., N.S., S.Cr., L.P.). Given the high number of papers retrieved during this procedure, we chose to first assess interrater reliability of eligibility ratings. Two pairs were made (S.Cl./N.S. and S.Cr/L.P) and both pairs of reviewers independently rated 100 papers based upon title, abstract and key words. As interrater reliability was excellent for both pairs (*k* = 0.96 to 1.00) (23), reflecting (almost) perfect agreement between both screeners, all references were divided and screened independently by the four reviewers. In case of doubt, a reviewer could consult the other reviewer of the pair to decide upon eligibility. Disagreements were solved by consensus (24). If there still was doubt about inclusion of the paper for the full text round (round 2), inclusion was discussed by all authors to find a consensus. In round 2, all possible eligible papers in the full texts round were screened by two independent researchers. We used the same procedure in case of disagreement as in round 1.

### 2.3 Data extraction

The first data extraction was performed on 23/08/2022. Data were extracted from the papers in two different ways. Firstly, a summary of findings was reported in a pre-designed table including the headings ‘objective’, ‘sample’, ‘stages and N’, and ‘conclusions’. This was done by N.S. and S.Cl. and supervised by H.V. Secondly, preliminary drafts were written for the result section according to a pre-designed format, including ‘full or partial staging model’, ‘reason for inclusion (criterion a, b, or c)’ and ‘main findings’. The various disorder sections were divided between N.S. and S.Cl. They composed summaries for each disorder section with assistance from L.P. and B.B. Each summary was supervised by one of the senior researchers (H.V., A.V., S.v.A., J.H., L.D.). Further details (objective, sample, stages and N and conclusions) of the included studies were reported in **Tables 1**–**9**.

### 2.4 Data analysis

**Figure 1:**
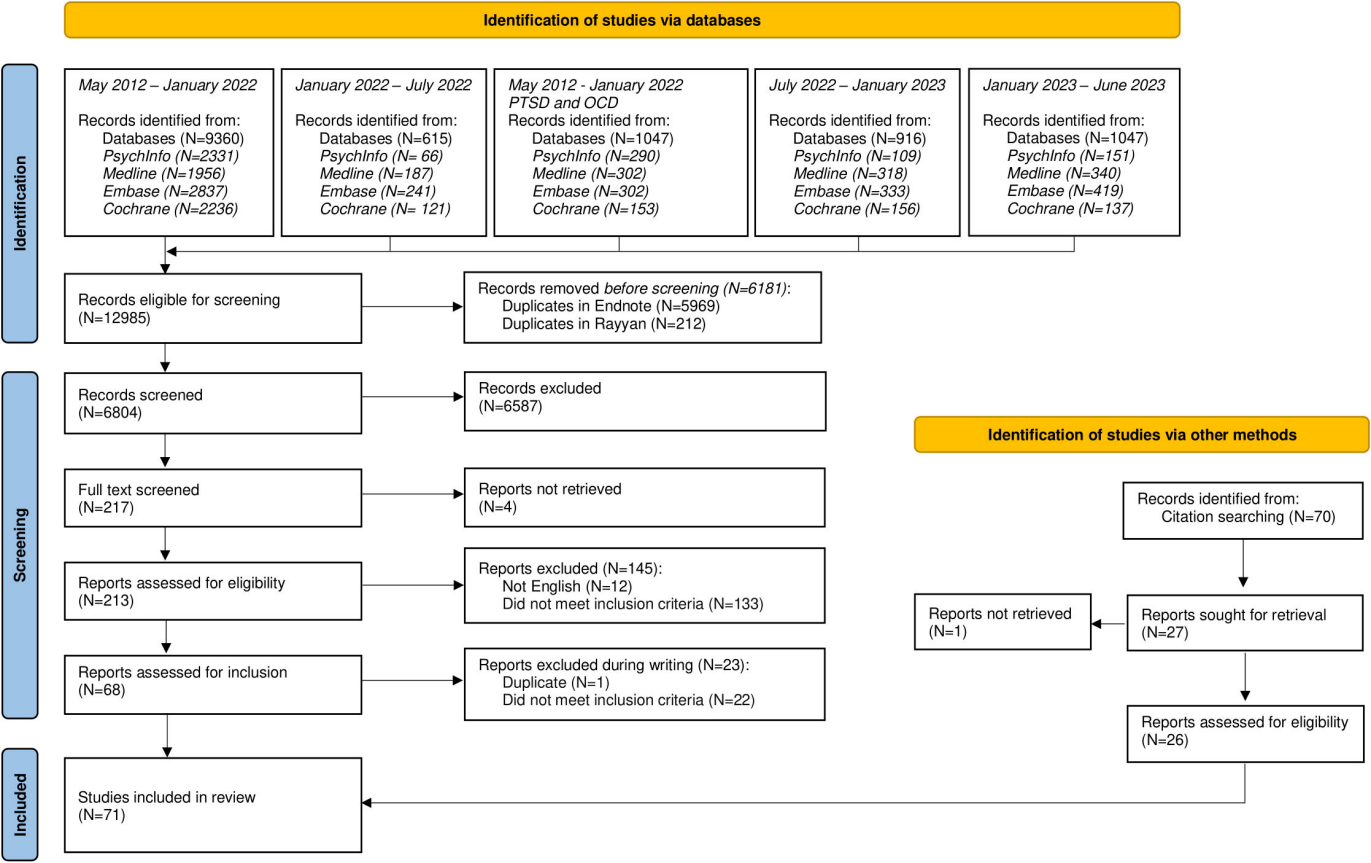
PRISMA flowchart.

The selection procedures and resulting outcomes are presented in a PRISMA flowchart (**Figure 1**). In line with procedures described for data extraction, we used the extracted data to summarize findings for each category of mental disorders. Data are presented in a table that summarizes key findings from the selected studies. In addition, brief text summaries describe the number of studies, their reasons for inclusion (i.e. whether they met inclusion criterion a, b, or c), whether the studies related to an existing staging model, the study objective, and a summary of findings.

## 3 Results

### 3.1 Selection of papers and study characteristics

Our search revealed 12985 papers. After removing duplicates, 6804 papers were screened on titles, abstracts, and keywords. Of these, 217 papers were subject to full text screening, of which 45 papers were included. Inspection of the reference lists of these 45 papers revealed another 26 papers that met inclusion criteria. The PRISMA flowchart is shown in **Figure 1**.

### 3.2 Schizophrenia spectrum and other psychotic disorders

Twenty-one papers met our inclusion criteria for schizophrenia or related psychotic disorders. One paper was included because of a theoretical focus on staging (‘category a’ see full definition in method section), seventeen papers focused on clinical characteristics of different stages (‘category b’) and three papers comprised interventions for one or several stages of a staging model (‘category c’). An overview of papers with a summary of aims and conclusions can be found in **Table 1**.

**Table 1:** Schizophrenia spectrum and other psychotic disorders.

Article	Objective	Sample	Stages and N	Conclusions
McGorry model based				
**Armando et al.** (25)	Theoretical and expansion to therapeutic interventions	Not applicable	0: psychotic like experiences 1a: attenuated psychotic symptoms 1b: brief self-limiting psychotic symptoms 2: FEP 3: Chronic psychosis	- Adding impairment of social function across the stages - Description of stage-appropriate MBT adaptations: mentalising enhanced prevention (stage 0), psycho-education and MBT for adolescent and families (1a; 1b), antidepressant and anxiolytics use (1b), group MBT and antipsychotics (2), adjusted MBT (3).
**Berendsen et al.** (26)	Cross-sectionally construct validity by symptomology and treatment response (HoNOS).	N=258 Admitted patients	2: FEP (N=48) 3b: recurrence or relapse after symptomatic remission (N=100) 3c: multiple relapses and incomplete remission (N=81) 4: chronic, severe, persisting or unremitting illness (N=29)	In later stages: - More severe negative symptoms, psychotic episodes in last year and therapeutic incompliance. - Less work and daily activities and support of close relatives. - Worse living situation and pre-morbid functioning. No difference on HoNOS scores across stages.
**Berendsen et al.** (27)	Clinical validity of splitting FEP based on DUP.	N=291	2a: FEP (<1 year DUP; N=38) 2b: FEP (>1 year DUP; N=24) 3a: incomplete remission from FEP (N=9) 3b: recurrent psychosis after symptomatic recovery (N=127) 3c: multiple relapses/incomplete remission (N=75) 4: chronic, severe, persisting or unremitting illness (N=18)	- Negative symptoms more severe in 2b than 2a with no other differences between these substages. - In later stages more hallucinations, negative and cognitive symptoms, older age and more often schizophrenia diagnosis. No difference in GAF scores or gender.
**Berendsen et al.** (28)	Cognitive performance associated with stages and transition at 3 and 6 years follow-up.	N = 927 at start (661 at 3 year and 547 at 6 year follow-up)	2a: FEP complete remission and GAF>70 2b: FEP incomplete remission 2c: FEP currently psychotic and GAF<70 3a: single relapse 3b-1: multiple relapses, symptomatic remission 3b-2: multiple relapse, currently psychotic 4: chronic, severe, persisting or unremitting illness (chronicity factor 5 or 6)	- At 3 vs. 6-year follow-up 242 vs. 159 patients remained stable in the stages, 94 vs. 77 improved and 259 vs. 261 declined. - Processing speed and working memory are significant associated with clinical stage, but not with stage-transition at 3 and 6 year follow-up. - antipsychotic medication, age and education level are significantly different between stages.
**Godin et al.** (29)	Clinical validity and stability of stages.	N=770 at start (297 at 1 year follow-up)	2a: FEP (<1 year DUP; N=89(32)) 2b: FEP(>1 year DUP; N=272(105)) 3a: incomplete remission from FEP (N=241(96)) 3b: recurrent psychosis after symptomatic recovery (N=112(50)) 4: chronic, severe, persisting or unremitting illness (N=56(14))	- In later stages: lower educational level, medication adherence, cognitive performance and QoL; more depressive, manic and extrapyramidal symptoms; multiple or classic antipsychotic use and chlorpromazine. -No difference in anxiety disorder, intoxications and metabolic function. - Follow-up at 1 year shows 92 patients remaining in stage 2, 41 progressing to stage 3 and 4 patients to stage 4. In stage 3 85 patients were stable, 42 improved to stage 2 and 19 progress to 4. Eight patients remain in stage 4.
**Kommescher et al.** (30)	Coping styles in different stages.	N=110	0–1: CHR (N=39) 2: FEP (N=19) 3 + 4: MEP (N=52) 3: incomplete remission and/or relapse(s) 4: severe and persistent state of illness	- More maladaptive coping styles in both CHR and MEP. - Mixed positive and negative coping styles in FEP.
**Li et al.** (31)	Stability of PNOS compared to SZ	PNOS:N =54 SZ=321	2 FEP	PNOS: - high drop-out (N=17), change to SZ diagnosis (N=8) - better treatment response and more remission, shorter DUP and DOI, more suicidality SZ: more negative symptoms
**Higuchi et al.** (32)	Clinical staging as covariate in CFA with MIMIC modelling	N=700	2 FEP (N=81) 3 MEP (N=342) 4 TRP (N=269)	Clinical stages and not DOI or age of onset predicts PANSS factor’s means. Drug-naïve FEP have higher PANSS means than MEP; TRP higher negative and disorganised factor than MEP.
**Ruiz-Iriondo et al**. (34)	RCT: IPT+EMT vs. TAU.	N=77 IPT+EMT=42 TAU=35	4: chronic Schizophrenia	- IPT+EMT has high compliance - TAU has a high drop-put (N=11) and hospitalisation (N=3) rate. - No significant differences between groups in improvements on cognition, social functioning, symptomatology and QoL
**Peralta et al.** (33)	Development, construct, outcome and predictive validity of a staging model and applying it to FEP participants with a 21 year follow-up.	N= 510 at start (243 at follow-up)	2a: single episode with full remission 2b: multiple episodes with full remission 3a: episodic with partial and stable remission 3b: episodic with partial remission and progressive 4a: chronic and stable 4b: chronic and progressive	- Most clinical variables progress in severity across stages with good construct validity. - Predictive validity variables had medium to large effect sizes, especially for developmental delay, childhood adversity, premorbid adjustment and cognitive reserve, mode of onset and negative symptoms. - The best predictor of staging is neurodevelopmental delay.
Other model				
**Bagney et al.** (37)	Relationship between negative symptoms and executive functioning according to DOI.	N=200	< 5 yrs: short DOI (N=42) 6-20 yrs: intermediate DOI (N=92) >20 yrs: long DOI (N=64)	- PANSS positive scores significantly differ between groups with higher scores in the intermediate and long DOI stage. - WSCT performance not significantly different between groups. - Negative symptoms correlate with WSCT categories completed in all groups, perseverative errors only in the long DOI stage and nonperseverative errors only with short DOI.
**Rapado-Castro et al.** (35)	RCT: role of DOI on response to NAC-treatment	N=121	<10 years (NAC: N=27; placebo: N=30) 10-20 years (NAC: N=21; placebo: N=18) > 20 years (NAC: N=11; placebo N=14)	Positive symptoms and functioning are improved by an interaction of NAC with DOI, especially with >20 years of DOI.
**Ortiz et al.** (38)	Explorative study: Clinical and psychopathological differences across stages.	Inpatients N=203	2: FEP with acute symptoms and subsequent exacerbations (N=53) <5 years DOI(both 3: residual phase between episodes 4: chronic illness with persistent symptoms) (N=28) ≥5 years DOI (both 3 and 4; N=122)	FEP: More remission compared to <5 and >5 years DOI. (62.3% vs. 35.7% and 34.4%; P = 0.002) and lower treatment resistance rates (15.1% vs. 42.9% and 45.1%; P = 0.001). No difference in early response or to clozapine. MEP: More positive, disorganized and hostility symptoms. No difference in negative or depressive symptoms.
**Sauvé et al.** (39)	Review (47 individual studies) of point prevalence negative symptoms.	N=10.021	1: UHR (N=1838) 2: FEP (N=2945) 3: yMEP (N=4939) 4: oMEP (geriatric or elderly) (N=299)	Avolition, alogia, asociality, blunted affect decrease from UHR to FEP but increase (together with anhedonia) from FEP to yMEP.
**Carrion et al.** (40)	Clinical validity RAP4 staging model, conversion rates and medication use over 3 years.	N=171	1: CHR- (N=46) 2: CHR+Mod (N=53) 3: CHR+Sev (N=39) 4: SLP (N=33)	- Conversion rate is higher and faster for later stages. - Low rates for CHR- group is no starting point for prodromen. - Interventions minor in CHR- and CHR+Mod; on symptomology in CHR+Sev and SLP. - Antidepressants in CHR- and CHR+Mod possibly prevents conversion rates.
**Dragioti et al.** (41)	Factor analysis on PANSS to determine stages with also focus on gender differences	Stable outpatients Male N=108 Female N=62	Early: 18-34 years Middle: 35-44 years Advanced: (≥45 years	Whole sample gave six factors and each individual stage a seven-factor structure with different factors dominant: - ‘Early’ has two depression and one delusional hostility factor ‘Middle’ has two residual negative disorganisation factors ‘Advanced’ a neurocognitive factor–Females more anxiety and depressive symptoms; Men more general pathology.
**Fountoulakis et al.** (42)	PANSS and illness duration by staging and gender differences.	Stable patients N=2358 Male N= 1429 Female N=929	1: first 3 years of duration 2a: 3-6 years 2b: 6-12 years 3a: 12-18 years 3b: 18-25 years4a: 25-40 years 4b: ≥ 40 years	Progression through the stages of PANSS. Factors: positive symptoms (PO), excitability/hostility (EH), negative symptoms (NE), depression/anxiety (DA) and neurocognition (Ncog). - ‘1’PO are dominant and EH- rises; - ‘2’ EH dominant and increasing, while PO decreases and stabilises. NE, DA and Ncog start to increase with DA dominant at stage end. - ‘2a’runs till DA is dominant over PO - ‘2b’ PO and EH stabilise, while NE, DA and Ncog rise. - ‘3 DA is dominant and DA, NE and Ncog rise until Ncog exceeds DA - ‘3a’ EH declines until NE, PO and Ncog are more prominent and ‘3b’ starts. - ‘4’Ncog rises exponentially, while other symptoms decline (decrease in DA = 4a; Ncog dominant and EH rising = 4b). - No difference in sexe in all results.
**Fountoulakis et al.** (43)	Development of PANSS clinical dimensions through illness stages.	N=2358	1 first 3 years 2a 3-6 years 2b 6-12 years 3a 12-18 years 3b 18-25 years 4a 25-40 years 4b ≥ 40 years	Positive symptoms dominating the first stage, excitement and hostility the second stage, depression and anxiety the third stage, and neurocognitive impairment the last stage. Negative symptoms are mostly stable during the stages with a mild increase from stage 3b onwards.
**Fountoulakis et al.** (44)	Role of gender, age of onset and DOI.	N=2358 Male 1429/female 929	Same as Fountoulakis et al., 2021	Age of onset 26.16 ± 8.07 yrs (males 25.08 ± 7.27/females 27.81 ± 8.92) and DOI 11.05 ± 10.93 yrs (males 11.03 ± 10.66/females 11.09 ± 11.36) with a significant effect for stage, onset (or duration) group and their interaction. No interaction of gender, age at onset, and DOI to influence the long-term course of schizophrenia.
**Falkai et al.** (36)	Efficicay and safety of cariprazine in pooled data.	N= 874	Early (<5 years and multiple episodes) (n=460) Late (≥15 years) (n=414)	- Early stage is dominated by positive and hostility symotoms with negative symptoms also present; late stage has more negative and cognitive symptoms present. - Cariprazine is more effective versus placebo in all stages, but has a larger treatment response in the early stage. - Cariprazine mostly reduces positive, negative, cognitive and hostility symptoms Insomnia and akathisia most prominent side-effects in both groups (cariprazine vs. placebo).
**Fusar-Poli et al.** (45)	Lived experience of patients	Not applicable	Premorbid Prodromal First episode Relapsing Chronic	First person accounts from patients and family members enriches the stages improving their validity. These accounts serve to grasp the dialectical dimension of psychosis and should be used in the model.

CFA, confirmatory factor analysis; CHR, clinical high risk patients; CHR-, clinical high risk with negative type symptoms; CHR+Mod, clinical high risk with mild to moderate positive symptoms; CHR+Sev, clinical high risk with severe positive symptoms; DA, depressive and anxiety symptoms; DOI=duration of illness; DUP, duration of untreated psychosis; EH, excitement and hostility; EMT, emotional management therapy; FEP, first episode psychosis patients; HoNOS, Health of the Nations outcomes scales; IPT, integrated psychological therapy; MBT =mentalization based treatment; MEP, multiple episode psychosis patients; MIMIC, multiple indicators multiple causes; NAC, N-acetyl cysteine; Ncog, neurocognitive symptoms; NE, negative symptoms; oMEP, older multiple episode patients; PANSS, positive and negative syndrome scale; PNOS, psychotic disorder not otherwise specified; PO, positive symptoms; QoL, Quality of Life; RAP, Recognition and Prevention Program; SLP, stage like psychosis with only positive symptoms of psychotic intensity; SZ, schizophrenic patients; TAU, treatment as usual; TRP, treatment resistant psychosis patients; UHR, ultra-high risk patients; WSCT, Wisconsin Card Sorting Test; yMEP, younger multiple episode psychosis patients.

Ten papers referred to McGorry and colleagues’ (4–6) model or to an adaptation of this model. Armando and colleagues (25) proposed an extension of McGorry’s model, by including associated impairments in social functioning in the formulation of stages. In addition, they described several stage-appropriate adaptations, based upon mentalization-based treatment. Eight papers investigated clinical features related to McGorry’s staging model. Berendsen et al. (26) supported construct validity by demonstrating worse affective, catatonic, and negative clinical markers in later stages. Although their paper revealed no difference in treatment response between stages, they found that duration of untreated psychosis (DUP) was associated with more symptomatology both at baseline and at two-year follow-up. In a follow-up paper, they proposed subdividing stage 2 in less (2a) versus more (2b) than one year of DUP, which was supported by their finding that negative symptoms were more severe in stage 2b than in stage 2a (27). In a subsequent paper, working memory and processing speed as measures of cognitive performance were found to be lower in later stages at baseline, however this was not the case anymore at 3 and 6 years of follow-up (28). Godin et al. (29) studied stage stability and validated their model by using number of episodes, daily functioning, and total scores on the Positive and Negative Syndrome Scale (PANSS). A subdivision of stages 2 and 3 based upon mood and cognition was proposed by data-driven cluster analyses. Kommescher et al. (30) found more maladaptive coping styles in high-risk populations (stage 0-1) and in stage 4. Li et al. (31) focused on stability of Psychotic disorder Not Otherwise Specified (PNOS) and suggested a subdivision of stage 2 in first episode PNOS (2a) or schizophrenia (2b). Another validation paper (32) yielded significant differences in the factor structure of the PANSS between stages independent of age of onset and duration of the illness, indicating staging may serve as an appropriate model to deal with the clinical heterogeneity of schizophrenia. Peralta et al. (33) focused on stages from the first psychotic episode onwards and made alterations to the McGorry model based on stability and progression of non-remitting illness. Validation of this adjusted model followed mainly from differences between stage 2 and 3A and addressed the factors involved in full versus incomplete remission. Finally, one paper investigated stage-tailored interventions referring to McGorry’s model. Ruiz-Oriondo et al. (34) found that combining integrated psychological therapy (IPT) with emotional management therapy (EMT) improved clinical symptoms, cognitive performance, social outcome, and quality of life in stage 4.

The remaining eleven papers departed from other staging models than McGorry’s. Nine papers studied correlates of stages and two articles [i.e., Rapado-Castro et al. (35), and Falkai et al. (36)] studied a corresponding intervention. Duration of illness was used in a paper by Bagney et al. (37) to define treatment groups and to study executive functioning related to negative symptoms. Rapado-Castro et al. (35) found a significant interaction between duration of illness and response to N-acetyl cysteine (NAC) for positive symptoms and functional variables, but not for negative or general symptoms. Specifically, this mediator effect for DUP in response to treatment was more evident in subjects with 20 years or more DUP, suggesting a potential advantage of adjunctive NAC on positive symptoms in patients with chronic schizophrenia. Ortiz et al. (38) used the four-stage model proposed by Cosci and Fava (11) to study clinical and psychopathological differences. Sauvé et al. (39) also used a four-stage model to study negative symptoms by categorising data from 47 studies on point prevalence. Carrion et al. (40) described and clinically validated a partial four-stage model that focused on the prodromal phases of psychosis as well as on conversion rates between stages. Dragioti et al. (41) developed and described a clinical staging approach using the PANSS pyramidal model and arbitrarily chose stages based on age groups and gender, as duration of illness was not available. Fountoulakis et al. (42) used duration of illness as the primary factor to describe stages and plotted illness duration against PANSS, factors resulting in four major stages (43). Positive symptoms were dominant in the first three years, excitement and hostility in the period between 3 and 12 years, depression and anxiety after 12 years, and neurocognitive impairment after 25 years. Negative symptoms were found to be mostly stable during all stages, with a mild increase from after 18 years onwards. Fountoulakis et al. (44) also studied the role of gender, age at onset (four groups) and duration of illness (seven groups) on the course of schizophrenia. They found a later onset and more benign course of the disorder for females, a relation between early onset and slower progression of the disorder for both sexes, while they did not find an effect of the disorder duration. Falkai et al. (36) used an early (<5 years illness duration) and late (≥ 15 years) stage to study the use of cariprazine versus placebo. Cariprazine was found to be more effective than placebo in both stages with a larger treatment response in the early stage. Lastly, Fusar-poli et al. (45) conducted a qualitative paper, with input from patient experts, and themes were discussed between patients, family members and professionals to design enriched stages, improving their validity, based upon the lived experience of patients.

### 3.3 Bipolar and related disorders

Twenty papers met inclusion criteria for bipolar and related disorders. Four papers presented theoretical proposals for a staging model, ten papers described clinical features of stages, five papers studied stage-related treatments and one paper (46) studied both clinical features and a stage-related intervention. Four different staging models, or adaptations, and associated interventions were mentioned, and eight papers used correlates of stages. See **Table 2**.

**Table 2:** Bipolar and related disorders.

Article	Objective	Sample	Stages and N	Conclusions
Duffy model based				
**Duffy et al.** (49)	Risk of SUD in high-risk offspring.	N=211 (88 male/123 female)	0: well 1: non-mood disorders (anxiety, sleep, ADHD, SUD) 2: minor mood disturbance 3: depression/major mood disturbance 4: mania (BD)	Significant risk of SUD in stage 1, 2 and 4 compared to 0. No difference between stage 1 through 4. Advise for psycho-education in patients and families on SUD and BD.
**Duffy et al.** (50) **(The developmental trajectory of BD)**	Risk of lifetime psychopathology in high-risk offspring and effect of parents lithium response on this risk.	N=229 HC: 86	0: well (and cognitive, mood and socialization symptoms in LNRP offspring) 1: non-mood disorders with anxiety and sleep problems (and ADHD, cluster A traits and learning disabilities in LNRP offspring) 2: minor mood disturbance 3: depression/major mood disturbance 4: BD I and II (and psychotic spectrum in LNRP offspring)	No long-term response on lithium in the parent gives more neurodevelopmental and psychotic disorders in offspring. Anxiety in childhood gives more major mood disturbances in adolescence. Advice is to take a detailed family history, put emphasis on preventive treatments and include lithium in the staging model.
**Duffy et al.** (51) **(Towards a comprehensive clinical staging model.)**	Review	Not applicable	Classical episodic BD(vs. spectrum BD) 0: well 1: non-specific syndromes (and developmental disorders) 2: minor mood and single episode depressive disorder (negative syndrome) 3: recurrent major depressive disorder (attenuated psychotic syndrome) 4A: classical BD(mixed-mania, psychotic/cyclic mania) 4B: BD with residual symptoms (psychotic disorders)	Proposal of an integrative model considering the high heritability of classical BD, importance of family history and taking into account childhood risk syndromes and neurodevelopmental disorders.
McGorry model based				
**Berk et al.** (52)	Narrative review updating evidence on staging and interventions.	Not applicable	0: asymptomatic with increased risk 1a nonspecific features of mood disorder 1b threshold features 2 first episode of mood disorder 3a recurrence of subthreshold symptoms 3b first threshold relapse 3c multiple relapses 4 persistent unremitting disorder	Interventions generic in stage 0/1a (psycho-education, self-help interventions), more intensive in 1b/2 (case-management, targeted psychopharmacotherapy and psychotherapy) and 3/4 (intensified interventions, relapse-prevention strategies).
**Chanen et al.** (53)	Integrating early intervention for BPD and mood disorders	Not applicable	0: asymptomatic with increased risk 1a nonspecific features of mood or BPD 1b threshold features 2 first episode of mood or BPD 3a recurrence of subthreshold symptoms 3b first threshold relapse 3c multiple relapses 4 persistent unremitting disorder	Generic interventions in early stages with HYPE program for BPD and psychosocial treatment for BD and unipolar depression.
**Power et al.** (54)	Review on early interventions in BD	Not applicable	0 high risk 1 prodrome/ultra-high risk 2 first manic episode 3 relapse 4 chronic course	Interventions suggested: genetic counseling (stage 0), psychoeducation (1), psychopharmacology (lithium, valproate or atypical antipsychotics alone or in combination) in combination with psychotherapy, family interventions and psycho-education (2), relapse prevention (3) and medication and psychosocial interventions with clozapine and electric convulsive therapy (4).
**Lee et al.** (55)	Retrospective evaluation of progression through the stages in BD during 5 years after onset.	N=136 (62 BD I/74 BD II)	2 first episode of mood disorder 3a recurrence of subthreshold symptoms 3b first threshold relapse 3c multiple relapses 4 persistent unremitting disorder	Progression through earlier stages is slow, while transition is faster in later stages. Transition of 40,3% from 2 to 3 and 12.7% from 3 to 4. BD-II has more transition to 4 (22.9% vs. 3.9% in BD-I). Transition rates are promoted by earlier age of onset, shorter DOI, older age at medication start, poor lithium response and unemployed. Comorbid OCD or BN progress more often to 3c and 4.
Other model				
**Van der Markt et al.** (56)	Applicability of a staging model and progression through the stages in 5 years after onset of BD.	N=99	0 increased risk 1 non-specific psychiatric symptoms or depressive episode(s) 2 first episode 3 recurrence 4 persistent unremitting illness	Average age of reaching the BD diagnosis (stage 2) was 29 years with 72% of people reaching stage 3 and 13% reaching stage 4 within 5 years. About 8% of patients went back to stage 3 after reaching stage 4
**Rosa et al.** (60)	Functioning and neurocognitive performance in staging.	N=43 HC N=54 BD	1 euthymia without psychiatric symptoms 2 psychiatric comorbidity/residual symptoms 3 marked cognitive and functional impairment 4 unable to self-care and live autonomously	From stage 2 onwards functional impairment is greater for BD than HC in a progressive fashion. Neurocognitive performance was poor in stage 3 and 4.
**Goi et al.** (61)	Empirical differences of pharmacological maintenance treatment between stages.	N=129	0 latent with high risk 1 euthymia without psychiatric symptoms 2 psychiatric comorbidity/residual symptoms 3 marked cognitive and functional impairment 4 unable to self-care and live autonomously	Number of mood episodes and psychiatric comorbidity is lower in earlier stages, while employment is higher. Monotherapy was more common in stage 1, clozapine in stage 4 and typical antipsychotics in stage 2 and 4.
**Van der Markt et al.** (62)	Comparison of two staging models on clinical utility and in between association.	N = 1396	Staging models conform: - Berk et al., 2007 with an adaptation to subdividing 3c in ≤5, 6-10 and >10 episodes (n=1218) Kapczinski et al., 2009 (n=1050)	- Most participants (n=1079) in the Berk model clustered in 3c with most distinctive parameter ‘number of episodes and in stage II (n= 396) and III (n=451) in the Kapczinski model with most distinctive parameter ‘the ability to work’. - There was a low association between both models: 0.21 (P< 0.05). For both models there is a significant change over the stages of age at onset, episode acceleration and treatment resistance.
**Macellaro et al.** (63)	Retrospective association between clinical markers of disease progression and stage increase in 4 staging models.	N = 100 (BD I = 53; BD II = 47)	Staging models conform (but without subclasses): - Berk et al., 2007 - Kapczinski et al., 2009 - Kupka and Hillegers, 2012 - Duffy, 2014	- All staging models show stage increases in ten years follow-up. - Mean stage increase was greater in patients with lower educational level(Berk model); in older patients, with lower educational level and no stressors at baseline (Kapczinski model); with younger age, duration of illness shorter than 25 years and duration of untreated illness shorter than 5 years (Kupka); with lower age at first depressive and elevated period and at first mood stabilizer (Duffy model) Lower stage increase is associated with BD II, no hospitalization, depressive onset and predominant polarity, less than 3 lifetime episodes, older age at first mood stabilizer (>40 years), shorter duration of illness (<25 years), engaged and employed status.
Correlates of stages				
**Rosa et al.** (64)	Functional outcome and clinical differences between patients with first- and multiple-episode BD	N=119 (NoE 1: n=60; NoE ≥ 2: n=59	NoE: 1 NoE: ≥ 2	Better functioning in first episode vs. multiple episodes. Depressive symptoms induce poor recovery at 6-12 months with multiple episodes.
**Magalhães et al.** (46)	Clinical aspects of stages based on number of episodes.	N=3345	<5 number of episodes 5-10 number of episodes >10 number of episodes	With increasing number of episodes there are more disabilities, chronic and severe symptoms, and less quality of life.
**Peters et al.** (65)	Effect of illness course and age of onset on recovery (time) and response to treatment.	N=205	1-9 NoE 10-20 NoE >20 NoE	NoE (depressive) and DOI are indicators of recovery rate and time. Intensive psychotherapy was associated with more likely and faster recovery compared to collaborative care in patients with less previous episodes and shorter duration of disease.
**Morris et al.** (66)	Effect of group psychoeducation vs. peer support on time to next episode.	N=304 (N=153 group; N=151 peer support)	1-7 NoE 8-19 NoE ≥20	Time to next episode is shorter with more than 8 previous episodes. No significant differences between groups.
**Murray et al.** (67)	Feasibility, potential effectiveness, and any negative effects of ORBIT in an open pilot trial.	N=26	Late stage (>6 NoE)	Online delivery of mindfulness-based psychological therapy for late stage BD appears feasible and effective, and ORBIT warrants full development
**Kamali et al.** (68)	clinical features associated with staging and predominant polarity (e.g., age of onset, type of mood episode atNu the onset of illness, use of antidepressants and antipsychotics) and measures of burden of illness (e.g., suicide attempts, substance use, and anxiety comorbidity)	N=482	Self-reported lifetime and past-year NoE	Staging and polarity are correlated, but do not change the effect of lithium or quetiapine The value of the number of mood episodes is less for staging and clinical prediction once multiple episodes have passed.
**Reinares et al.** (69)	Identifying clinical, functional and cognitive predictors to specify prognostic subtypes.	N=106	1 good outcome 2 poor outcome	Episode density (number of episodes divided by disease duration), residual level of depressive symptoms, verbal intelligence and inhibitory control are significant predictors of subtypes. While age, age of onset and duration of illness are not.
**Grande et al.** (70)	Using clinical variables to classify patients to stages.	N=115	Early Late	Cluster analysis on number of episodes, age at onset, time elapsed since the first episode and FAST scores give significant differences between early and late stages. Early stage has fewer episodes, older age at onset and better functioning.

ADHD, attention deficit hyperactivity disorder; BN, bulimia nervosa; BD, bipolar disorder; BPD, borderline personality disorder; DOI, duration of illness; FAST, functioning assessment fast test; HC, healthy controls; HYPE, Helping Young People Early program; NoE, number of episodes; LNRP, lithium non-responsive parent; OCD, obsessive compulsive disorder; ORBIT, online, recovery-focused, bipolar individual therapy; SUD, substance use disorder.

Two studies focused upon clinical characteristics, based on a previously proposed clinical staging model of Duffy and colleagues (47, 48). The first study found that, regardless of bipolar parent substance abuse, offspring in stage 1, 2 and 4 were likely to develop substance use disorder compared to stage 0, while no further significant differences were found between stages 1 to 4 (49). The second study found that offspring of one parent diagnosed with bipolar disorder with no long-term response to lithium were more likely to develop psychotic and neurodevelopmental disorders compared to offspring with a lithium responding parent. Therefore, they advised including parents’ response to lithium in the staging model (50). A subsequent conceptual paper distinguished between classic episodic and spectrum bipolar disorder within the staging model and underlined the high heritability of the disorder and the need for a thorough family history assessment to add context to early (otherwise nonspecific) risk syndromes (51).

Berk et al. (52) described an adaptation of McGorry’s staging model of psychotic and severe mood disorders (4) for bipolar disorder, which was also used by Chanen et al. (53) in a combined model for borderline personality disorder (BPD), unipolar depression and bipolar disorder given their common underlying risk factors, age of onset, co-occurrence and overlapping core symptoms (see personality disorders, below). The same model was then slightly simplified, and interventions tailored to each stage were proposed (54). A third paper used the model of Berk et al. (52) to study clinical features. Illness progression was monitored during five years after onset of bipolar I or II disorder with slower progression through the earlier stages and faster transition in the later stages (55).

Van der Markt et al. (56) refined a combined model based on the staging models of Berk et al. (57), Kupka and Hillegers (58) and Duffy et al. (51). Retrospective life charts were used to study progression through stages in the five years after onset of bipolar disorder (stage 2) with the majority reaching stage 3 and a smaller amount reaching stage 4. Two papers referred to the staging model described by Kapczinski et al. (59). Rosa et al. (60) evaluated functional and neurocognitive performance across stages and found a progressive impairment in functioning along the stages with neurocognitive decline in the later stages. Goi et al. (61) studied patterns of pharmacological treatment with more functional impairment when more medication was used from the second stage onward. Two papers compared different staging models with each other. Van der Markt et al. (62) compared the models of Berk et al. (57) and Kapczinski et al. (59) and found a low association suggesting the models act complementary. Furthermore, they suggested using ‘dispersion over the stages’ to assess the clinical utility of a model. Macellaro et al. (63) also compared these models with the models of Kupka and Hillegers (58) and Duffy (51) and retrospectively found stage progression in all four models during a ten year period. Results showed that an increased number of mood episodes worsened severity of next episodes and reduced treatment response.

Finally, several papers discussed correlates of stages for bipolar disorder. Most studies used ‘number of episodes’ as a proxy to define stages. One study (64) studied functional outcomes in patients with only one versus more (depressive, manic, or mixed) episodes. First episode patients were younger and more autonomous, had less cognitive complaints and better work performance, and were better able to enjoy spare time and their companions. Magalhães et al. (46) observed more severe symptoms and a lesser quality of life after more episodes had passed. In a subset of (chronic) patients, antidepressant use was evaluated with no significant difference between subgroups.

Four papers studied differential effects of interventions for bipolar disorder. Peters et al. (65) found that intensive psychotherapy was associated with more likely and faster recovery compared to collaborative care in patients with less previous episodes and shorter duration of disease (i.e., earlier stage). Similarly, Morris et al. (66) noted more beneficial treatment effects for structured group psychoeducation compared to optimized unstructured group support in patients with fewer episodes. Murray et al. (67) defined the late stage as more than six episodes and found that online mindfulness therapy improved quality of life. This contrasts with findings of Kamali et al. (68) who found that self-reported number of lifetime and past-year mood episodes predicted neither the effects of lithium nor quetiapine. However, illness duration was on average more than 23 years and number of episodes high. Reinares and colleagues (69) proposed still another partial staging model of remitted patients and used latent class analysis to find a two-class model fitting the data best. Classification in ‘good’ or ‘poor’ outcome stages was based on functional outcome with episode density (number of episodes divided by disorder duration), residual level of depressive symptoms, estimated verbal intelligence and inhibitory control as strongest predictors. Grande et al. (70) used cluster analysis on several clinical variables (number of episodes, age of onset, time since first episode and functioning) to divide their study population in an early and late stage with the first functioning better, having a later age of onset and fewer episodes.

### 3.4 Depressive disorders

Our search revealed one newly proposed staging model including a study on clinical correlates, two papers on clinical correlates of existing staging models and one stage-related treatment paper. A more extensive description of findings can be found in **Table 3**.

**Table 3:** Depressive disorders.

Paper	Objective	Sample	Stages and N	Conclusions
McGorry model based				
**Verduijn et al.** (72)	Validity of clinical staging in MDD and 2-year progression.	N=2333 (baseline) N=2012 (2-year follow-up)	0 no depressive complaints, family history of MDD (n = 287;264) 1a mild or nonspecific symptoms (n=116; 100) 1b moderate but subthreshold symptoms (n=834;748) 2 first episode (n=230;191) 3a incompletion remission of first episode (n=129;96) 3b recurrence or relapse (n=127;110) 3c multiple episodes (n=394;340) 4 severe, persistent or unremitting MDD (n=216;163)	- Poorer scores on clinical characteristics (depression severity, duration, anxiety and disability) form a linear pattern across the stages, with better scores in 3b than 3a, and provide evidence for construct validity. - At follow-up 517 patients have MDD with more cases, larger disability scores and more time spent with depressive symptoms in higher stages, except for a decline in 3b. More chronic stages (3a, 4) have poorer characteristics than relapsing stages (3b, 3c). - No significant difference was found between consecutive stages (2, 3a, 3b, 3c, 4) and similar scores were found for stages only differing in number of episodes (2,3b, 3c).
Hetrick model based				
**Reneses et al.** (73)	Correlates of Hetrick’s 2008 model with severity of depression, disability and resistance to treatment and to test a modification on the model	N=133	2: (N=29) 3a: (N=13) 3b1: (N=51) 3b2: (N=23) 3c: (N=10) 4 (N=7)	- GAF score were significantly different between 4 and all other stages. - CGI scores were significantly different between 4 and 2; 4 and 3a; 4 and 3b; 4 and 3c; 3a and 3c. - Resistance to treatment measured by MSM was significantly different for: 2 and 3b2; 2 and 3c; 2 and 4; 3b1 and 3b2; 3b1 and 3c; 3b1 and 4. - No significant differences were found between stages, the HAM-D total score and SDS. - Significant correlations were found between MSM, GAF, CGI and stages. - No significant correlations between HAM-D, SDS and depression stages.
Other model				
**Rhee et al.** (71)	Characteristics among behavioural problems and comorbidities in persons in different MDD stages.	N=8053	1: new onset of MDD (past year but not prior; N=509) 2: chronic or unrecovered MDD (past year and prior; N=3871) 3: recovery from MDD (prior but no past-year; N=3673)	- Recovered adults compared to new onset MDD are: older, male, no minority, not married or uninsured, no pain or multiple medical comorbidities, more likely to have a history of (lifetime) treatment for MDD, suicide attempts and recovery from multiple but no current psychiatric disorders among alcohol use disorder and had a higher income and education. - Recovered adults compared to chronic MDD are: older, no minority, not married or uninsured and had a higher income and education, less likely to have a history of lifetime treatment for MDD, suicide attempts as well as antisocial or borderline personality disorder and more likely recovered from other, but have no current, psychiatric or medical disorders. Recovery was more likely from alcohol use disorder, but no current substance use disorder. - Chronic versus new Onset MDD are: older, no minority, more likely to be a veteran and have a history of lifetime treatment for MDD, suicide attempts, recovery from multiple and also current psychiatric and medical disorders among borderline personality disorder as well as more education. There was no difference in substance use disorders.
**Dodd et al.** (75)	Study associations between number of previous depressive episodes and treatment response.	N=5627	- Without previous episodes (N=1381) - With at least one previous episode (N=4246) - With ≤3 previous episodes (N=3930) - With > 3 previous episodes (N=1697).	No significant differences were found between number of previous depressive episodes and treatment response

MDD, Major Depressive Disorder; MSM, Maudsley Staging Method; GAF, Global Assessment of Function; CGI, Clinical Global Impression; HAM-D, 17 items-Hamilton Depression Scale; SDS, Sheeham Disability Scale.

Rhee and colleagues (71) proposed and studied a staging model for Major Depressive Disorder (MDD) with three stages, namely stage 1 ‘new onset of MDD’ (past-year but not prior MDD), stage 2 ‘chronic or unrecovered MDD’ (past-year and prior MDD) and stage 3 ‘recovery from MDD’ (prior but no past-year MDD). Verduijn and colleagues (72) studied the construct and predictive validity of a clinical staging model for MDD, as proposed by McGorry and colleagues (4), consisting of different stages (0, 1A, 1B, 2, 3A, 3B, 3C, 4) based on the severity and duration of symptoms and the number of episodes. At 2-year follow-up, most of the clinical characteristics, such as severity and duration of symptoms and disability, worsened across the stages. Reneses and colleagues (73) modified Hetrick and colleagues’ model (74), by separating those with “recurrence from a previous depressive episode that was stabilized with a complete remission” into stage 3b1. They found that ‘treatment resistance’ – operationalized using the Maudsley Staging Model based upon a) disease episode duration, b) symptom severity and c) treatment failures – distinguished best between clinical stages and they argued that this variable differentiates best the stages in Hetrick and colleagues’ (74) model. Finally, a study by Dodd and colleagues (75) found no significant differences between the number of previous depressive episodes and treatment response.

### 3.5 Anxiety disorders

The two papers identified refer to clinical correlates (see **Table 4**). Clarke and colleagues (76) used Hickie and colleagues’ (77) (see below) transdiagnostic model to classify participants according to the stage of their anxiety disorder. They found that clinical features, such as greater psychological distress, higher levels of depression, and more alcohol use distinguished participants in stages 2 and 3 from participants in stage 1b. There were no differences in reported levels of anxiety between stage 1b and the higher stages. Bokma and colleagues (78) adapted Hickie and colleagues’ (77) and McGorry and colleagues’ (4) models, differentiating between ‘subclinical’ stages (0, 1a, 1b), clinical stages with comorbidity (2b, 3b, 4b) and without (2a, 3a, 4a) comorbidity. Linear trends were found with severity of anxiety, depression and disability increasing in the later stages.

**Table 4:** Anxiety disorders.

Article	Objective	Sample	Stages and N	Conclusions
McGorry and Hickie based				
**Clarke et al.** (76)	Application of the staging model of Hickie et al. (2013) for social anxiety disorder	N=143 (13-30yrs)	1b: attenuated syndrome (N=53) 2 or above: discrete disorder (N=22)	- Clinical features like more psychological distress, higher levels of depression, and more alcohol use distinguished subjects in 2 and 3 from subjects in 1b. - There were no differences in reported levels of anxiety between 1b and higher stages. - 143 participants (13-30 years) with anxiety disorders (71.33% met criteria for social anxiety disorder, 8.39 panic disorder, 6.29% generalized anxiety disorder and 5.59% major depressive disorder). The majority (70.63%) had multiple diagnoses. Using several sources of information, the authors assigned 140 participants to a stage: 8.57% to stage 1a, 68.57% to stage 1b, 19.29% to stage 2, 3.57% to stage 3. Clinical features like more psychological distress, higher levels of depression, and more alcohol use distinguished subjects in stage 2 and 3 from subjects in stage 1b.
**Bokma et al.** (78)	To assess the validity of staging to anxiety disorders	N=2352	- ‘Subclinical’ stages: 0: asymptomatic no anxiety, at risk (N=574) 1a: help-seeking, mild to moderate anxiety (N=371) 1b: attenuated syndromes subthreshold anxiety (N=170) - Clinical stages with comorbidity: 2b: discrete disorders (N=268) 3b: intermittent or persistent (N=246) 4b: chronic, severe (N=314) - Clinical stages without comorbidity 2a: discrete disorder (N=159) 3a: intermittent or persistent (N=95) 4a: chronic, severe (N=155)	The model of clinical staging for anxiety disorder has construct and predictive validity.

### 3.6 Obsessive-compulsive and related disorders

Our search found two conceptual proposals and one study of clinical correlates (see **Table 5**). Fineberg and colleagues (79) proposed a partial staging model, starting with subthreshold symptoms that may represent the first at-risk stage for obsessive compulsive disorder (OCD), for which they suggest psychoeducation for parents on not accommodating of the obsessive-compulsive symptoms. They additionally proposed an ultra-high-risk group, defined by at-risk symptoms *and* vulnerability because of environmental risk factors. The next stage includes patients who do meet the syndromal threshold for OCD, for which they recommend cognitive behavior therapy and medication (SSRI). Finally, they describe later stages as consisting of patients who appear to be resistant to treatment.

**Table 5:** Obsessive-compulsive and related disorders.

Article	Objective	Sample	Stages and N	Conclusions
**Fineberg et al.** (79)	A consensus statement on evidence for early intervention of OCD, i.e. a proposal of a staging model	Not applicable	- At risk stage: subthreshold symptoms ultra-high-risk group, defined by at-risk symptoms and vulnerability because of environmental risk factors - Full blown OCD - Treatment resistant OCD	A staging model could set the stage for validating of stages, associated clinical correlates and treatment
**Fontenelle and Yücel** (80)	Proposal of a staging model for OCD, based on a review	Not applicable	- 0: healthy individuals who do not report any OCD symptoms 0a: but do have family history of OCD or tics 0b: environmental risk factors 0ab: both - 1 (ultra high risk): subthreshold symptoms, a family history of OCD or tics and/or environmental risk factors - 2: mild to moderate symptoms 2a: first episode 2b: multiple episodes or chronic - 3: severe symptoms	Use of a staging model could be relevant for identification and management of OCD, especially for subthreshold OCD.
**Benatti et al.** (81)	Application of the staging model of Fontenelle and Yücel (2019)	N=198	At baseline: 1 (Y-BOCS score 1-13) (N=10) 2 (Y-BOCS score 14-34) (N=52) 3 (Y-BOCS score 35≥) (N=8)	70 patients diagnosed with OCD were included to study this staging model. Baseline allocations were 14,3% to stage 1, 74,3% to stage 2 and 11,4% to stage 3. At follow-up (12 months) 27.1% was assigned to stage 1, 67.1% to stage 2, and 5.7% to stage 3. Between baseline and follow-up, 67,1% remained unchanged, 24,3% improved to a lower stage and 8,6% deteriorated to a worse stage. The worsened group displayed a higher prevalence of comorbid disorders, a higher prevalence of somatic obsessions and more unfavorable employment features. The improved group showed higher rates of magical thinking and violence/harm obsessions.

OCD, Obsessive-compulsive disorder; Y-BOCS, Yale-Brown Obsessive-Compulsive Scale (Y-BOCS).

Fontenelle and Yücel (80) proposed a staging model for OCD, based upon a review, considering clinical markers, potential biomarkers, and outcome characteristics. They proposed stage 0, 0a, 0b, 0ab, 1, 2, 2a, 2b and 3. The authors identify different types of symptoms, levels of family accommodation, and comorbidity for these stages and they also propose different treatment strategies. This model was studied by Benatti and colleagues (81) in a follow-up study. About two-thirds remained unchanged. The worsened group displayed a higher prevalence of comorbid disorders, a higher prevalence of somatic obsessions and more unfavorable employment features. The improved group showed higher rates of magical thinking and violence/harm obsessions.

### 3.7 Trauma and stressor-related disorders

Three conceptual papers and one study of correlates (see **Table 6**) were identified. McFarlane and colleagues (82) distinguished between five stages of posttraumatic stress disorder (PTSD). Their model describes a range of possible neurobiological changes associated with different stages and they stress how these biological processes may also affect the immune system and underpin somatic comorbidities. A proof-of-concept study used machine-learning techniques to predict stage assignment, based upon McFarlane’s staging model (83). The authors found that number of symptoms, the Clinician-Administered PTSD Scale-DSM-5 edition (CAPS-5) total score, global severity score, and presence of current/previous trauma were most predictive of PTSD stage. Nijdam et al. (84) proposed to extend McFarlane’s model with information processing systems and psychophysiological stress and emotional reactivity, and consciousness. The authors proposed interventions for each stage of the disorder based upon their extended approach. The search also retrieved a hypothetical staging model for persistent complex bereavement disorder (PCBD) following the loss of a loved one in traumatic circumstances (85), to inform treatment decisions.

**Table 6:** Trauma and stressor-related disorders.

Article	Objective	Sample	Stages and N	Conclusions
**McFarlane et al.** (82)	Proposal of a staging model to the biological mechanisms of PTSD and treatment	Not applicable	0: trauma exposed asymptomatic but at risk 1a: Undifferentiated symptoms 1b: subsyndromal distress 2: first episode of full-threshold symptoms 3: Persistent symptoms - 3a: incomplete remission of first episode - 3b: recurrence or relapse - 3c: multiple relapses or worsening following incomplete treatment response 4: severe unremitting	Provides a template to differentiate between biological underpinnings of PTSD in different stages and to examine different biological models for PTSD and their overlap.
**Ramos-Lima et al.** (83)	A proof-of-concept study to examine the viability of a predictive model (machine learning) for staging PTSD	N=122	Based but merged, model of McFarlane et al., 2017: 1: full diagnosis not reached 2: first episode 3: persistent symptoms 4: severe	Most predictive of PTSD stage: - Number of symptoms; - Total score on CAPS-5; - Global severity score; - Presence of current/previous trauma.
**Nijdam et al.** (84)	Proposal of extended staging model and recommendations of staged-interventions, based on McFarlane (2017)	Not applicable	See the model above of McFarlane (2017)	While the model of McFarlane (2017) was based on possible neurobiological markers of stage, each stage in the model of Nijdam is extended with: - Information processing systems - Psychophysiological stress and emotional reactivity, and consciousness Besides, the authors suggest to define grades of treatment resistance, as another phenomena than stages in the course of the disorder. Hypothesis of staged interventions are described in the article.
**Boelen et al.** (85)	To introduce a staging, profiling and stepped care model for PCBD	Not applicable	0: exposure to bereavement with acute symptoms 1a: undifferentiated symptoms 1b: subtreshold PCBD 2: first episode 3: persisting symptoms 4: unremitting PCBD with increasing chronicity and comorbidity	Provides a template to differentiate between biopsychosocial underpinning of PCBD in different stages and to match treatment.

PTSD, Posttraumatic Stress Disorder; PCBD, Persistent Complex Bereavement Disorder; CAPS-5, Clinician-Administered PTSD Scale-DSM-5 edition.

### 3.8 Feeding and eating disorders

Three papers on staging models for feeding and eating disorders were identified, two conceptual papers and a study of clinical correlates (see **Table 7**). Treasure et al. (86) proposed a staging model, based upon a systematic review, distinguishing between four stages (high risk, early, syndrome, severe enduring). They focused on the progression of anorexia nervosa (AN), as few data were found to support staging models for bulimia nervosa and binge eating disorders. Stages for AN were largely based upon the presence of predisposing features, illness features and neuroprogressive features, and duration of illness. The authors propose different interventions with so-called ‘psychoprotective’ (cognitive dissonance strategies and social media literacy) and ‘neuro-protective’ (promoting healthy eating and physical activity) interventions for the high-risk stage, and family support and family-based therapy for the early stage. A slightly modified version of the same model was included in Treasure et al. (87), differentiating ‘high risk’ in a ‘high risk’ and an ‘ultra-high risk/prodrome stage’. The authors explore different interventions for the severe and enduring anorexia nervosa (SE-AN) stage, based upon a maintenance model including interpersonal consequences, behavioral consequences, and reactions to chronic stress. Their suggestions include caregiver skill training, habit reversal therapy, exposure techniques, neuromodulation, and pharmacotherapy. The clinical utility of a concept of SE-AN was further explored in an outpatient service (88). Results highlighted different courses and service use associated with different stages of AN.

**Table 7:** Feeding and eating disorders.

Article	Objective	Sample	Stages and N	Conclusions
**Treasure et al.** (86)	To examine evidence for a staging model for eating disorders, based on a systematic review	Not applicable	High risk Early Syndrome Severe enduring	Early stages include illness features on top of predisposing features, while syndrome and severe enduring stages involve gradually more neuroprogressive features consequential to prolonged starving or abnormal eating patterns. Stages are also distinguished based upon duration of illness, with early stages including illness features for less than three years, while severe and enduring AN (SE-AN) includes illness features for at least seven years. Staging is useful in providing prognostic information and to match interventions.
**Treasure et al.** (87)	To describe factors of the Enhanced Cognitive Interpersonal Maintenance Model, that contribute to the development of SE-AN	Not applicable	High ED risk Ultra-high risk/Prodrome Early stage illness (<3yr) Full stage illness Severe and enduring illness	Enhanced Cognitive Interpersonal Maintenance Model: - Interpersonal consequences - Behavioural consequences - Chronic stress
**Ambwani et al.** (88)	To explore the clinical utility of SE-AN	N=187	Early stage (<3yr) (N=60) SE-AN (>7yr and DASS >=60) (N=41)	SE-AN reported more lifetime hospitalizations and worse eating disorder symptoms, and work and social wellbeing compared to early stage.SE-AN reported fewer symptomatic changes over time compared to early-stage, especially in relation to work and social adjustment.

AN, Anorexia Nervosa; SE-AN, Severe Enduring – Anorexia Nervosa; ED, Eating Disorder.

### 3.9 Disruptive, impulse-control, and conduct disorders

No papers were found for staging models in conduct disorders.

### 3.10 Substance-related and addictive disorders

No staging models were found for substance use disorders.

### 3.11 Personality disorders

Two conceptual papers on staging models were found for borderline personality disorder (BPD) (see **Table 8**). Chanen et al. (53) (2016) presented a combined model for BPD and mood disorder, based upon their similar age of onset, common risk factors, frequent co-occurrence and overlapping clinical phenomenology. This model further outlines a mental health care response to each stage. Interventions are more generic and simpler at stage 0 and 1a and become more specialist and intensive at stages 1b and 2, and further on. A second staging model for BPD has been proposed by Hutsebaut et al. (89). Their model uses features of BPD, co-occurring psychopathology, and psychosocial disability as criteria to distinguish between five stages.

**Table 8:** Personality disorders.

Article	Objective	Sample	Stages and N	Conclusions
**Chanen et al.** (53)	Integrate early intervention for BPD and mood disorders, presentation of a staging model	Not applicable	0: increased risk 1a: Mild or nonspecific symptoms 1b: subthreshold features 2: first episode 3a: recurrence of subthreshold symptoms 3b: first threshold relapse 3c: multiple relapses 4: persistent, unremitting disorder	- BPD in young persons appears a reliable and valid disorder. - BPD and mood disorders do have features in common. - There is a need for early intervention in BPD.
**Hutsebaut et al.** (89)	To provide a conceptual framework for BPD with a focus on assessment and treatment	Not applicable	0: problems in self-regulation and interpersonal functioning, without clinical diagnosis, but with potential effects on school and social functioning 1: subthreshold BPD features, co-occurring presenting symptoms of mental state disorders and imminent developmental arrest 2: a first episode of full BPD with significant problems in the four core areas of BPD (affect, impulse control, identity, and interpersonal functioning), co-occurring mental state disorder and moderate to severe psychosocial impact 3: longer duration of BPD or recurring episodes of (partial) remission and relapse, associated with persistent and severe mental state disorder(s) and severe and chronic impairments in social and vocational functioning 4: chronic BPD, multiple co-occurring mental state disorders and lack of participation in social and professional life	The authors propose specific interventions related to each stage, starting with school-based prevention, early intervention programs (Stage I) and adolescent-adapted BPD interventions (Stage II), to standard BPD treatment (Stage III) and long-term supportive interventions (Stage IV).

BPD, Borderline Personality Disorder.

### 3.12 Transdiagnostic staging models

The thirteen included papers all studied clinical correlates and are outlined in **Table 9**. Twelve papers used Hickie and colleagues’ (77) transdiagnostic staging model. In a study of young people seeking mental health care, assignment to stages was reliable and at follow-up 11%, 19%, and 33% of respective stages 1a, 1b, and 2 progressed to a later stage. In a large-scale longitudinal observational study, Iorfino and colleagues (90) investigated the transition rates from a help-seeking stage and an attenuated syndrome stage to a discrete disorder stage in patients with anxiety, mood, and psychotic disorders. Stage progression from the help-seeking stage was associated with older age, self-harm, manic-like experiences and lower social functioning, and engagement in education, employment, or training. Progression from the attenuated syndrome stage was associated with older age, psychotic-like experiences, previous use of psychiatric medication and a history of childhood psychiatric disorders. Addington and colleagues (91, 92) followed youth with emerging severe mental illness (SMI), assigned to similar stages. Of the help-seeking participants, 50% remained symptomatic, and 7.5% moved to the next stage or developed a SMI. Of the attenuated syndrome participants, 9% developed a SMI and one-third had symptom remission within 12 months. Hermens and colleagues (93) used the same staging model in a sample of young people seeking mental health care for psychotic and/or depressive symptoms. They found that the discrete disorder group displayed the most impaired neuropsychological profile (i.e., problems in memory and executive functioning), compared with the attenuated syndrome group, with a healthy control group being least impaired. To further inform empirical research in transdiagnostic staging, Ratheesh et al. (94) explored interrelationships and risk factors among mood, psychotic and anxiety 1b symptom stages. Anxiety, depressive and psychotic stages were found to be inter-related and associated with the following risk factors: sex at birth (female), more emotional and behavioral difficulties in early adolescence, and life events in late adolescence. Hypomania was not interrelated with these stages nor with these risk factors. Based on these findings, the authors suggested that anxiety, psychotic and depressive symptoms could form a combined transdiagnostic stage in their studied cohort.

**Table 9:** Transdiagnostic.

Article	Objective	Sample	Stages and N	Conclusions
McGorry and Hickie based				
**Hickie et al.** (77)	Characteristics and progression through stages in persons 12-30 years.	N=209	1a: help-seeking (N=21) 1b: attenuated syndrome (N=112) 2: discrete disorder (n=53) 3: persistent or recurrent disorder (23)	Progression through stages: - 1a to: 1b (N=2) - 1b to: 2 (N=19); 3 (N=1) - 2 to: 3 (N=16) Diagnoses were bipolar disorder (N=3), psychotic disorder (N=3) and syndromal transfomations of known depressive disorders (N=14).
**Iorfino et al.** (90)	Characteristics change with progression through stages 1a-2 in persons 12-25 years.	N=2254	1a: help-seeking (N=685) 1b: attenuated syndrome (N=1370) 2: discrete disorder	Progression through stages: - 1a to: 1b (N=253); 2 (N=18) based on older age, lower social functioning, engagement in education, employment or training, manic-like experiences and self-harm. - 1b to: 2 (N=176) associated with older age, psychotic-like experiences, previous use of psychiatric medication and a history of childhood psychiatric disorders. Bipolar syndrome accounted for N=86; psychotic disorders for N=47 and anxiety/depressive disorders for N=61 persons who transitioned to stage 2.
**Addington et al.** (91)	Characteristics in staging for persons 12-25 years at risk for SMI.	N=243	0: asymptomatic (N=43) 1a: help-seeking (N=52) 1b: attenuated syndrome (N=108) * HC (N=42)	- No clinical difference between HC and stage 0. - Beliefs about oneself, ruminations and anhedonia did not differ between 1a and 1b - 1b group had higher clinical complaints compared to the other stages and HC
**Addington et al.** (92)	Progression through stages in 12 months for persons 12-25 years at risk for SMI	N=243	0: asymptomatic (N=41) 1a: help-seeking (N=53) 1b: attenuated syndrome (N=107) 2: discrete disorder * HC (N=42)	Progression through the stages: - Both HC and 0 to: 1a (N=1) and 1b (N=2) - 1a to: 1b (n=2); 2 (N=2) and remission (N=13) - 1b to: 2 (N=9) and remission (N=34)
**Hermens et al.** (93)	Association of stages with differential neuropsychological impairment patterns in persons 18-30 years.	N=194	1b: attenuated syndrome (N=94) 2/3: (N=100) 2: discrete disorder 3: persistent or recurrent disorder *HC (N=50)	- Neuropsychological profile is significantly worse (p<0.05) in 2/3 group (z scores 0.0- -1.0) than HC (0.0-0.5) and intermediate in 1b (0.0- -0.5). The same applied to subsamples of 1b (n=79) and 2/3 (n=41) with a mood syndrome but without psychosis. - Antipsychotic or mood stabilizer use was three times higher in 2/3 group while antidepressant use was comparable with 1b. - Depressive and psychotic symptoms are only correlated with neuropsychological profile in 1b
**Ratheesh et al.** (94)	Interrelationships and risk factors among mood, psychotic and anxiety 1b symptom stages	Data of 3629 participants of the Avon Longitudinal Study of Parents and Children (ALSPAC) cohort study	Stage 1b: - Significant symptoms of psychosis - Significant symptoms of hypomania - Moderate to severe depression - Moderate to severe anxiety	Descriptive methods and network analyses were used to examine overlap among the four different symptom stages. Anxiety, depressive and psychotic stages were inter-related, and associated with the following risk factors: sex at birth (female), more emotional and behavioral difficulties in early adolescence, life events in late adolescence. Hypomania was not interrelated with these stages and neither with these risk factors.
**Romanowska et al.** (95)	1.Describe neurocognitive functioning of youth (12-25 yr) at risk of SMI 2.Compare neurocognitive functioning of stage 0, 1a and 1b, and HC	N=243	0: asymptomatic (N=41) 1a: help-seeking (N=52) 1b: attenuated syndrome (N=108) *HC = 42	Differences between stages: - 1a performed worse than HC regarding speed of processing, working memory, reasoning, problem solving and overall neurocognitive functioning. - 1b showed lower processing speed and poorer working memory than 0. - 0 performed worse in working memory, reasoning, and problem solving, than HC.
**Tickell et al.** (96)	Validation of neuropsychological profile patterns in persons 16-30 years in a larger sample and with longer follow-up compared to Hermens et al. (2013).	Baseline (N=497) Follow-up (N=170)	1b: attenuated syndrome (N=262; FU N=92) 2/3 (or 2+): discrete disorder (N=235; FU N=78): 2 (M=156; FU N=58) 3 (N=79; FU N=20)	- Neuropsychological profile is significantly worse (p<0.05) in 2/3 group (z scores -0.1 to -1.0) compared to 1b (0.2 to -0.6) except for processing speed, sustained attention, visual working memory, cognitive flexibility and verbal fluency (all groups z score <-0.5). - Persons showing up for follow-up had a significantly younger age of onset and showed improvement except on verbal learning, verbal memory, cognitive flexibility and verbal fluency in the 2+ group.
**Scott et al.** (97)	Association between sleep-wake cycle patterns and stages for persons 12-30 years.	N=154	1a: help-seeking with mild symptoms (N=18) 1b: attenuated syndrome (N=82) 2+: established mental disorder (N=54) *HC = 21	- Frequency of delayed sleep schedules during weekdays were similar in HC and 1a. But increased progressively at 1b and 2+. Same pattern was found for weekends. - 1b had later sleep onset on weekends than HC. 1b and 2+ had later sleep offset on weekdays and weekends. - Stage 1b and 2+ had later sleep offset on week and weekend nights compared to HC. - Wake after sleep onset was higher in all stages than in HC. - Sleep efficiency was lower in 1a and 2+ than in HC. - Older age, medicated status and later weekdays sleep offset were the strongest predictors of later stages.
**Cross et al.** (98)	Clinical and demographic factors related to appointment adherence in persons 12-25 years.	N=828	1a: help-seeking and no current (DSM-IV) diagnosis (N=378) 1a: help-seeking with a current (DSM-IV) diagnosis (N=201) 1b: attenuated syndrome and no current (DSM-IV) diagnosis (N=105) 1b: attenuated syndrome with a current (DSM-IV) diagnosis (N=144)	- Number of made appointments was not correlated with attendance rate. Age, psychological distress and functioning were correlated with both. More appointments were made and attendance was better with higher psychiatric severity at 1b with a diagnosis, while half the number in 1b without a diagnosis. - Men made more appointments but adherence worsened with progressing stage. Attendance in females was related to functioning level, not to disease severity or stage.
**Hamilton et al.** (99)	Use of health care attributes per stage in young people with different mental disorders.	N = 412 clinicians	1b: attenuated syndrome 2: discrete disorder	- Beneficial attributes most frequently varied by *disorder*: Family engagement and participation; collection and processing of biological samples; neurocognitive assessment and symptom tools; information exchange with other services; face to face consultations at home; case management with multidisciplinary team. - Beneficial attributes most frequently varied by *stage*: neurocognitive assessment and symptom tools; delivery of care in subacute residential setting; collection and processing of biological samples; case management with a multidisciplinary team. - Stage, disorder and interaction between those, were predictors of clinician-perceived benefit of attributes. - Attributes perceived as relevant in all disorders (>70%): Information exchange with other service providers; family engagement and participation; face-to-face consultations in the room. - Attributes more relevant in stage 2 than 1a for the whole sample: Neurocognitive assessment and symptom tools; information exchange with other service providers; face to face consultations in the room and at home; case management with a multidisciplinary team; collection and processing of biological samples; family or caregiver peer support; young person peer support; care delivery in subacute residential setting.
**Hartmann et al.** (100)	Validate and refine CHARMS criteria	N=114	CHARMS- (i.e. 1a, the control group) CHARMS+ (i.e.1b)	- 46% of the CHARMS+ group met criteria for more than one at-risk mental state [high risk for psychosis (UHR), high risk for severe depression (HRD), high risk for mania (HRM), and high risk for borderline personality disorder (HRB)] - After 12 months, 34% of the CHARMS + group, as compared to 3% of the CHARMS- group, had transitioned to stage 2. When three or more at risk mental states were met, transition risk to stage 2 in the CHARMS+ group increased to 40%
Other model				
**Wigman et al.** (101)	Experience Sampling Methods to assess momentary mental states (PA, NA, paranoia) across stages	N=621 of the East Flanders Prospective Twin Study register	SCL-severity: Level 1 Level 2 Level 3 Level 4	- Interactions were found between SCL-severity and negative affect, positive affect and paranoia at *t-1*, and predicted negative affect at *t.* - interactions were found between SCL-severity and negative affect, positive affect and paranoia at *t-1*, predicting paranoia at *t.* - No significant interactions were found between SCL-severity and negative affect at *t-1*, positive affect, or paranoia, at *t-1*, predicting positive affect at *t.*

DSM, Diagnostic and Statistical Manual of Mental Disorders; FU, follow-up; HC, healthy controls; NA, negative affect; PA, positive affect; SMI, serious mental illness; *t*, time point.

Two papers investigated neuropsychological functioning across stages. Romanowska and colleagues (95) reported that participants in the help-seeking stage 1a performed worse than healthy controls on measures of speed of processing, working memory, reasoning, problem solving and overall neurocognitive functioning. Participants in the attenuated syndrome stage 1b showed lower processing speed and poorer working memory than participants in the asymptomatic stage 0. Participants in stage 0 performed worse than healthy controls in working memory, reasoning, and problem solving. Tickell and colleagues (96) found differences in verbal learning, verbal memory, visual memory and set shifting between stage 1b and 2+. Both groups showed similar improvement in neuropsychological functioning at follow-up.

In a study of disturbed sleep-wake cycle patterns, Scott and colleagues (97) found that help-seeking young people experienced more wake time after sleep onset, compared with healthy young people. Participants in the mild symptom and established disorder stages had lower sleep efficiency, compared with healthy young people. Delayed sleep increased across stages. Cross and colleagues (98) used the medical records of young people to assign them to one of four groups: stage 1a lacking a current (DSM-IV) diagnosis, stage 1a with a current diagnosis, stage 1b lacking a current diagnosis, or stage 1b with a current diagnosis. Age, gender, severity of illness, functioning and psychological distress had differential associations with both planned treatment intensity and attendance rates.

One paper used an online survey method to identify perceived utility of a wide range of attributes of mental health care for different disorders in the attenuated syndrome and discrete disorder stages (99). Hartmann et al. (100) introduced CHARMS (Clinical High At Risk Mental State), a pluripotent, at-risk mental state concept. CHARMS criteria are proposed as an extension of UHR (Ultra High Risk) criteria, extending these to psychosis (UHR), high risk for severe depression (HRD), high risk for mania (HRM) and high risk for BPD (HRB).

Finally, Wigman and colleagues (101) used experience sampling methods in a sample of women, 95% twins, to study the reciprocal impact of momentary mental states (positive affect, negative affect, and paranoia) over time across different stages of severity of psychopathology; staging was operationalized across four levels of increasing severity of psychopathology, based on the total score of the Symptom Checklist. They found that more severe stages were characterized by stronger connections and more variable connections between mental states. Moreover, severe stages indicated more individual-specific associations between mental states over time. These results suggest that, as individuals move through progressive stages, the dynamics between different mental states become increasingly stronger (referring to the nomothetic concept of staging), and the differences between individuals become progressively larger (referring to the idiographic concept of profiling).

## 4 Discussion

The current systematic review aimed to provide an overview of the past decade of clinical staging models for potentially progressive mental disorders included in DSM-5. Our second aim was to review empirical studies of the reliability, validity, and clinical utility of staging models published over this period. Our search identified 71 papers, with 13 newly proposed staging models or extensions on earlier staging models. We identified 58 empirical papers, 47 of which focused upon validating staging models by studying clinical correlates. Nine papers investigated treatment interventions based upon a model of staging. Two of the 58 empirical papers had several aims (studying correlates and interventions or proposing a staging model and studying correlates of the proposed model). One of the 71 papers focused on a newly proposed staging model combined for bipolar disorder and mood disorder (53) and is therefore described under both bipolar disorder and personality disorders. By far most papers were found for schizophrenia and related psychotic disorders (21 papers), and bipolar and related disorders (20), while other categories of mental disorders were much less represented: depressive disorders (4), anxiety disorders (2), obsessive-compulsive and related disorders (3), trauma and stressor-related disorders (4), feeding and eating disorders (3) and personality disorders (2). In addition, 13 papers used a transdiagnostic model. No papers were identified for disruptive, impulse-control and conduct disorders, or for substance-related and addictive disorders. Compared with the original Cosci and Fava review (that included 78 papers), fewer papers met the inclusion criteria for the current review. However, it is noteworthy that the current review excluded certain types of papers that were included in Cosci and Fava’s review. For example, papers referring to ‘stages of change’ in the treatment of substance use (SUD) or eating disorders were not included in the current review, as we conceived these ‘stages’ not as reflective of disorder progression. The current review identified no papers on SUD and only three on eating disorders that met the inclusion criteria.

The results of our search suggest that the field has been producing consistent outputs but that it is not growing rapidly. The review also shows some evolution in the field over the last decade. While models for psychotic and bipolar disorders still dominate the field, the current review identified additional staging models for specific syndromes, including obsessive-compulsive, trauma and stressor-related, and personality disorders, along with several papers using a transdiagnostic approach to staging.

Our first aim was to summarize new conceptual models of staging in psychiatry. Staging models were primarily developed to better capture the dynamic nature of the trajectories of psychopathology and the emergence of mental disorder syndromes over time, complementing the cross-sectional nature of traditional psychiatric classifications (2). Conceptually, most staging models share a similar design, resembling the disease staging of their medical counterparts. Most models consider individual disorders and use DSM-5 criteria to distinguish between stages: stage 1 is usually defined in terms of subthreshold diagnosis, while the demarcation with stage 2 is usually based upon a transition from subthreshold to full DSM-5 syndrome diagnosis. Similarly, transition to stage 3 is usually determined by recurrence, i.e., a new acute episode of the disorder. However, it is questionable whether this ‘disease-based’ approach, rooted in psychopathology, fits adequately with the typical heterogeneity of mental health issues and the unpredictable course of many mental disorders (102). This raises several issues.

Firstly, many DSM-5 diagnostic thresholds are arbitrary (103) and it’s questionable whether diagnostic criteria will ultimately enable valid distinctions between stages. Staging approaches fit better with diseases of which the underlying psychopathology is fully understood, including clear biomarkers for disease onset and progression (104). This is clearly not the case for mental disorders, limiting the validity of a disease-approach. In addition, unlike untreated physical illness which often progresses in a predictable way, mental disorders are often characterized by multifinality, and course and outcomes are more difficult to predict (105). Secondly, many severe mental disorders, including psychotic and personality disorders, might be characterized by unstable symptomatic episodes, that are accompanied by longstanding psychosocial consequences (106). Typically, psychosocial disability is considered to occur in parallel with, or be a *consequence* of, mental disorder. Arguably, for some conditions, especially personality disorder, psychosocial disability could be the source of the disorder (107), while for others, disability might precede disorder. Overall, some disabilities are more intrinsically interwoven with mental impairments, in contrast to psychosocial disabilities arising as outcomes of somatic medical conditions. Moreover, many severe mental disorders are characterized by a ‘symptom-disability gap’, in which psychopathological symptoms fade away, while psychosocial disability might be more persistent and influential upon the longer-term outcome of mental disorders (108). Two recent conceptual models include psychosocial disability to define distinction between stages, thereby deviating from a purely psychopathological approach (25, 89). Both models include psychosocial disability as a marker of more enduring stages of mental illness. Thirdly, nearly all staging models describe markers of *psychopathology* while ignoring areas of resilience that might mitigate the impact of a disorder. Indeed, especially in mental health, disorder progression might also be influenced by (lack of) domains of resilience and/or adaptive functioning (109, 110). Fourth, clinical staging highlights the importance of early detection and intervention and thus the relevance to identify symptoms and features that characterize a subclinical stage. A major issue given the heterogeneity of phenomena within this early stage, however, is to distinguish mild subclinical expressions of a disorder from early stages that may indeed progress into more severe manifestations. A key challenge therefore may be to identify symptoms that mark increased risk across different diagnostic categories with greater specificity. Finally, and probably most importantly, for disease staging to function as a prototype for clinical staging it must account for the heterotypic continuity of mental disorders (111) and heterogeneity among stages. Studies have repeatedly demonstrated that mental disorders are not fixed and independent entities but are highly correlated leading to differential expressions of psychopathology throughout the lifespan (112). For these reasons, models focusing upon single disorders are likely to be of limited explanatory value. Interestingly, despite our search terms being more likely to elicit disorder-specific staging models, we also retrieved several studies on transdiagnostic models. The number of trans-syndromal models required us to summarize these papers in a distinct and new category. This appears to reflect emerging recognition of the limitations of single disorder staging models and a shift toward the development of transdiagnostic or trans-syndromal models (113), exemplified by the first international consensus statement on transdiagnostic clinical staging in youth mental health (114). Such developments within the field of clinical staging align with a broader reconceptualization of psychopathology that stresses general dimensions or clusters of psychopathology as opposed to traditional nosologies, like the formulation of the p-factor (115), the emergence of HiTOP (116) and the revised alternative models of personality disorders (117). However, as opposed to these concepts, staging models emphasize more strongly the progressive nature of psychopathology. Still, in line with these developments, we contend that rather than rigidly adhering to single disorder models that are solely based in psychopathology, future development of clinical staging models will need to be more carefully tailored to incorporate evidence regarding the structure and development of psychopathology, along with other specific features of mental disorders that set them apart from their somatic counterparts. Staging models might also benefit from incorporating resilience and/or psychosocial functioning criteria that might be associated with (non-)progression. It remains to be seen whether these aims can be addressed while also achieving reliability and ease of use, which are crucial to clinical staging’s pragmatic aims.

Taking all these issues together, we believe that from a conceptual point of view, models of clinical staging may need to be tailored better to the complexities of mental disorders. Although it might affect their reliability and ease-of-use, it is worthwhile to consider whether staging models might benefit from considering more criteria related to resilience and/or psychosocial functioning that might be associated with (non-)progression.

Our second aim related to the empirical support for the validity and clinical utility of clinical staging in psychiatry. Our review found that the distinction between stages was validated in almost all reviewed papers, despite the abovementioned conceptual issues. These findings suggest that psychopathology can be approached as progressive with later stages being associated with increased severity, different symptom profiles, and more neurocognitive impairments across different studies. This underpins the notion that patients with late-stage disorders might indeed have different clinical needs, when compared with those with early-stage disorders. Importantly, outside the field of psychotic and bipolar disorders, empirical papers are largely lacking, suggesting that in most areas the focus is still on the conceptualization of models, with only initial efforts to validate them. Most papers investigated clinical correlates of different stages, while there were only ten publications in which treatment or intervention was based upon a staging model. Our search did not identify publications that explicitly investigated the clinical utility of clinical staging models, suggesting that these models have had only limited impact upon clinical practice. This might be due to the lack of a consensus about which model(s) to adopt in practice, along with the narrow scope of single syndrome models. If clinical staging models are to be used in routine clinical practice, there is a clear need for evidence supporting their direct utility. This requires more studies to establish the potential effectiveness and cost-effectiveness of treatment planning and interventions according to predetermined models of clinical staging. In the absence of sufficient studies demonstrating the clinical utility of models of clinical staging, their impact on clinical practice or on developing treatment guidelines can be expected to be rather limited.

### 4.1 Strengths and limitations

Notable strengths include using the same eligibility criteria as Cosci and Fava (11), allowing for comparison and identification of potential trends in publications about clinical staging. We also performed an *a priori* interrater reliability test, showing very high to almost perfect levels of agreement, to avoid differences among raters in selection of papers for eligibility. Our systematic review was also preregistered.

Limitations include introducing extra search terms referring to disorders for which we assumed a potentially lifelong progressive course, like personality and conduct disorder (that may progress into antisocial personality disorder). However, we did not include neurodevelopmental disorders in our search, such as autism spectrum disorder, and we excluded papers with a primary focus on neuroanatomy or biological markers. Furthermore, we chose to include extra search terms, i.e., PTSD and OCD, in a follow-up search to include categories of mental disorders that have been re-allocated into new individual categories in DSM-5. Although this inclusion was justified given by the transition to DSM-5, which occurred after the publication of Cosci and Fava’s review, this deviated from our preregistered search protocol. Furthermore, we deviated from Cosci and Fava’s (11)) eligibility criteria by excluding papers referring to stages of change in substance abuse and eating disorders. Also, we did not include papers referring to treatment-resistant depression, as we decided that such papers did not reflect the criterion of a partial or full staging model in our eligibility criteria. Finally, we did not perform any quality assessment of the designs used in the included studies in this review, which could be seen as a shortcoming. Although recommended, we believe the usefulness of such a quality assessment is limited given the aim of our systematic review to provide a broad overview of the current state of clinical staging and given the type of papers retrieved by the search. The results of our review revealed very heterogeneous studies, varying from RCT’s, cohort studies, longitudinal studies, mixed methods studies, survey studies and qualitative studies. Widely used tools for Risk of Bias analyses, such as Cochrane’s tool or ROBINS-I, are not suitable to report on the quality of heterogeneous studies. Therefore, we opted to merely describe findings, as a reflection of academic and clinical interest in staging. Consequently, no conclusions can be drawn about the quality of evidence derived from these studies. Our results should be interpreted largely as a reflection of efforts to validate staging models, rather than as justification of these models in and of themselves. reviews could rigorously assess the quality of evidence.

## 5 Conclusions

The current review demonstrates only a slow increase in interest in clinical staging as an alternative way of assessing the progressive course and severity of psychopathology. In addition, for most categories of mental disorders, staging models are still in a phase of conceptualization and initial validation. There is a general lack of (new) evidence supporting the clinical utility of clinical staging. Current models will need to be better tailored to the complexities of mental disorders, including taking a transdiagnostic or trans-syndromal approach, and including areas of resilience and psychosocial disabilities given, their complex relationships with psychopathology.

## Data Availability

The original contributions presented in the study are included in the article/**Supplementary Material**. Further inquiries can be directed to the corresponding author.

## Supplementary material

Supplementary Material 1 Search strategy used for PsycINFO.
