# Supplementary material for: The progress in the field of clinical staging for mental disorders within the last decade: an updated systematic review
Source: Front Psychiatry. 2025 Jan 15;15:1473051. doi: 10.3389/fpsyt.2024.1473051 (PMC11775755; doi:10.3389/fpsyt.2024.1473051)
Supplement: Supplementary Material 1 — Search strategy used for PsycINFO. [file Table1.docx]

Supplementary Material

# Supplementary Data

***Supplementary material***

*Search strategy used for PsycINFO*

Limits: Peer-reviewed journal, human, English, published between 1^st^ of May of 2012 to current

1. (Mental disorder.ti. or mental disorder.ab. or mental disorder.id.) AND (stage.ti. or stage.ab. or stage.id. or stages.ti. or stages.ab. or stages.id. or staging.ti. or staging.ab. or staging.id.)
2. (Psychiatric disorder.ti. or psychiatric disorder.ab. or psychiatric disorder.id.) AND (stage.ti. or stage.ab. or stage.id. or stages.ti. or stages.ab. or stages.id. or staging.ti. or staging.ab. or staging.id.)
3. (Mood disorder.ti. or mood disorder.ab. or mood disorder.id.) AND (stage.ti. or stage.ab. or stage.id. or stages.ti. or stages.ab. or stages.id. or staging.ti. or staging.ab. or staging.id.)
4. (Anxiety disorder.ti. or anxiety disorder.ab. or anxiety disorder.id.) AND (stage.ti. or stage.ab. or stage.id. or stages.ti. or stages.ab. or stages.id. or staging.ti. or staging.ab. or staging.id.)
5. (Substance abuse disorder.ti. or substance abuse disorder.ab. or substance abuse dirsorder.id.) AND (stage.ti. or stage.ab. or stage.id. or stages.ti. or stages.ab. or stages.id. or staging.ti. or staging.ab. or staging.id.)
6. (Schizophrenia.ti. or schizophrenia.ab. or schizophrenia.id.) AND (stage.ti. or stage.ab. or stage.id. or stages.ti. or stages.ab. or stages.id. or staging.ti. or staging.ab. or staging.id.)
7. (Eating disorder.ti. or eating disorder.ab. or eating disorder.id.) AND (stage.ti. or stage.ab. or stage.id. or stages.ti. or stages.ab. or stages.id. or staging.ti. or staging.ab. or staging.id.)
8. (Conduct disorder.ti. or conduct disorder.ab. or conduct disorder.id.) AND (stage.ti. or stage.ab. or stage.id. or stages.ti. or stages.ab. or stages.id. or staging.ti. or staging.ab. or staging.id.)
9. (Personality disorder.ab. or personality disorder.ti. or personality disorder.id.) AND (stage.ti. or stage.ab. or stage.id. or stages.ti. or stages.ab. or stages.id. or staging.ti. or staging.ab. or staging.id.)
10. (Obsessive compulsive disorder.ti. or obsessive compulsive disorder.ab. or obsessive compulsive disorder.id.) AND (stage.ti. or stage.ab. or stage.id. or stages.ti. or stages.ab. or stages.id. or staging.ti. or staging.ab. or staging.id.)
11. (Posttraumatic stress disorder.ti. or posttraumatic stress disorder.ab. or posttraumatic stress disorder.id.) AND (stage.ti. or stage.ab. or stage.id. or stages.ti. or stages.ab. or stages.id. or staging.ti. or staging.ab. or staging.id.)
